# *In vitro* aging behavior of dental composites considering the influence of filler content, storage media and incubation time

**DOI:** 10.1371/journal.pone.0195160

**Published:** 2018-04-09

**Authors:** Jörn Krüger, Reinhard Maletz, Peter Ottl, Mareike Warkentin

**Affiliations:** 1 Department of Prosthodontics and Material science, Faculty of Medicine, University of Rostock, Rostock, Germany; 2 Department of Material Science and Medical Engineering, Faculty of Mechanical Engineering and Marine Technology, University of Rostock, Rostock, Germany; Institute of Materials Science, GERMANY

## Abstract

**Objective:**

Over time dental composites age due to mechanical impacts such as chewing and chemical impacts such as saliva enzymes and food ingredients. For this research, the focus was placed on chemical degradation. The objective of this study was to simulate hydrolysis by using different food simulating liquids and to assess their impact on the mechanical parameter Vickers microhardness (MHV) and the physicochemical parameter contact angle (CA).

**Methods:**

Specimen of three composites (d = 6 mm, h = 2 mm; n = 435) classified with respect to their filler content (wt%), namely low-filled, medium-filled and highly-filled, were stored for 0, 14, 30, 90 and 180 days in artificial saliva (pH 7), citric acid (pH 3; pH 5), lactic acid (pH 3; pH 5) and ethanol (40%vol; 60%vol) and assessed regarding to MHV and CA. Statistics: Kruskal-Wallis test, stepwise linear regression, bivariate Spearman Rank Correlation (*p < 0*.*05*).

**Results:**

While stored in artificial saliva, acid and ethanol the CA decreased especially for the low- and medium-filled composites. It was shown that rising the filler content caused less surface changes in the CA. Storage in ethanol led to a significant decrease of MHV of all composites. Regression analysis showed that the effect of *in vitro* aging on MHV was mainly influenced by the composite material and therefore by filler content (*R*^*2*^ = *0*.*67; p < 0*.*05*). In contrast, the CA is more influenced by incubation time and filler content (*R*^*2*^ = *0*.*2; p < 0*.*05*) leading to a higher risk of plaque accumulation over time. Significance: *In vitro* aging showed significant changes on the mechanical and physicochemical properties of dental composites which may shorten their long-term functionality. In conclusion, it can be stated, that the type of composite material, especially rising filler content seems to improve the materials’ resistance against the processes of chemical degradation.

## Introduction

Dental composites have gained increasing importance and usage over the last several years, caused especially by rising demands on aesthetic restorations [1, 2, 3]. These dental composites mainly consist of a methacrylate based resin matrix filled with organic and inorganic particles of different shapes and sizes (macro-, micro- and nano-fillers). Many efforts continue to be made to optimize the chemistry of filler and matrix composition to provide enhanced clinical application and performance. For example, changes in filler size and content have improved the mechanical properties (e.g. flexure strength, fracture toughness, tensile strength, polymerization shrinkage, Vickers microhardness (MHV)) and enabled further application in posterior restorations [3, 4].

Besides the mechanical properties, the surface of a restoration is relevant to clinical performance. Plaque adhesion, which is relevant for caries and periodontitis, depends on among others parameters on the surface roughness and surface free energy [5, 6]. Due to strong chewing forces, wear resistance is another important clinical parameter [4], especially over the long operational lifespan of modern composites with annual failure rates of 2.4% after ten years [7].

All of these properties are influenced by filler content and the polishing procedure that is utilized [8, 9]. Studies have shown, that the changes of the initial state are of clinical interest, since chemical and mechanical degradation occur under *in vivo* conditions [10, 11, 12, 13]. Especially with regard to chemical degradation as it causes damage on the surface and subsurface, in particular on the resin-matrix, the filler and the matrix-filler interface [14]. As a consequence of degradation and the following erosion, material properties potentially decline over the long operational lifespan, which compromise function by increasing plaque adhesion and accelerating abrasion [15]. In general, the main reasons for replacement of composite fillings are still secondary caries and fractures of the restorations [7, 16, 17]. Therefore, degradation was considered apart from other factors (e.g. high caries risk) as a possible reason for a composite’s failure [1].

Several artificial aging procedures such as artificial saliva, acids or ethanol solutions have been used in studies to simulate these effects [18, 11, 14, 13, 19]. Along with the chemical effect of various food ingredients, like fruit acids, fatty acids and bacterial derived acid, saliva itself and enzymes may cause material softening [20, 21, 22]. Within this discussion, there must be a differentiation between the elution of uncured residual monomers (short term effect) and the elution of degradation products, which may occur even after an extended period of time.

The objective of this study was to investigate the degradation behavior of dental composites with varying filler rates, in various artificial aging solutions over a maximum incubation time of 180 days. This study evaluates based on the systematic method and the practical based selection of storage media the relative influence of three variables: (a) filler content, (b) storage media and (c) incubation time compared to each other and the initial state after insertion of the composite, as this has not yet been sufficiently analyzed.

## Materials and methods

### 2.1 Composites and specimen preparation

Three commercial available composites were chosen with respect to their filler content and been classified into low-, medium- and highly-filled groups by weight (wt%). 435 disc-shaped specimen (diameter 6 mm, height 2 mm) (details see Table 1) were prepared according to the manufacturer’s guidelines using silicone molds and polymerized with a light source (1.000 mW/ cm^2^, Celalux 2, VOCO GmbH, Cuxhaven, Germany).

**Table 1:** Composites chosen by class of filler weight.

Class by filler weight	Filler content		Filler particles	Resin matrix	Product name (Abbreviation)	Manufacturer	Manufacturer class
	wt%	%vol					
Low-filled	64	51	highly-dispersed silicon dioxide barium-/ strontium borosilicate (Ø 0.7 μm)	BisGMA, TEGDMA, UDMA	Arabesk Flow (AF) # 1131254	Voco GmbH, Cuxhaven, Germany	micro-hybrid composite
Medium-filled	77	56	microfillers (Ø 0.05 μm) barium-/ strontium borosilicate (Ø 0.7 μm)	BisGMA, TEGDMA, UDMA	Arabesk Top (AT) # 1218277	Voco GmbH, Cuxhaven, Germany	micro-hybrid composite
Highly-filled	89	73	silanized silicon dioxide (Ø 20–40 nm) glass ceramic fillers (Ø 1 μm)	BisGMA, BisEMA, TEGDMA	GrandioSO (G) # 1212204	Voco GmbH, Cuxhaven, Germany	nano-hybrid composite

The oxygen inhibition layer was removed by carbide finishing burs (H48LQ, Komet, Lemgo, Germany) under permanent water spray coolant for 30 s (20.000 rpm). They were polished with a rubber polisher (9524 UF, Komet, Lemgo, Germany) using permanent water spray coolant for 30 s (6.000 rpm).

### 2.2 Artificial aging

From each composite five randomly chosen specimen were grouped into one sample. Following evaluation of the initial state of contact angle (CA) and microhardness (MHV), samples were stored and artificially aged for 14, 30, 90 and 180 days. Seven different media were used: artificial saliva [AS], lactic acid [LA] (CAS number: 79-33-4, Carl Roth, Karlsruhe, Germany) (pH 3 and pH 5), citric acid [CiA] (CAS: 77-92-9, Carl Roth, Karlsruhe, Germany) (pH 3 and pH 5) and ethanol [ET] (CAS: 64-17-5, Carl Roth, Karlsruhe, Germany) (40%vol and 60%vol). Samples were incubated at 37°C (Inkubator 1000, Heidolph, Schwabach, Germany) under continuous agitation (Unimax 1010, Heidolph, Schwabach, Germany).

In the below paragraph, all seven different storage media are briefly detailed. Artificial saliva (according to DAC/NRF 7.5 Germany potassium chloride (CAS: 7447-40-7), sodium chloride (CAS: 7647-14-5), Disodium hydrogen phosphate dodecahydrate (CAS: 10039-32-4), Calcium chloride dihydrate (CAS 10035-04-8), Magnesium chloride hexahydrate (CAS: 7791-18-6), sorbic acid (CAS: 110-44-1), D-sorbitol (CAS: 50-70-4)) was used as a reference to simulate oral conditions without any sour influence [23]. Lactic acid (pH 3 and pH 5) was chosen as a metabolite of oral plaque to assess influence of surrounding bacteria. Citric acid (pH 3 and pH 5) was used to simulate fruit acid and ethanol, which is a well-known artificial aging media and equivalent to fatty acids and food ingredients [24, 18, 14, 11, 13]. Both acids were diluted with artificial saliva to the desired pH values, which were measured with a pH-Meter (FiveEasy^™^ pH, Mettler Toledo, Giessen, Germany). The ethanol solution was diluted with distilled water. After incubation, all samples were dried 24 hours under a vacuum using silica gel in a desiccator. Afterwards, they were stored under dry and dark conditions until measurement. To avoid saturation by degradation products the storage media of every sample was refreshed completely after every 4 weeks.

### 2.3 Contact angle and surface free energy

Contact angle (CA) was measured by using the sessile drop method (OCA20, DataPhysics Instruments GmbH, Filderstadt, Germany). The samples surface was carefully cleaned with isopropanol (CAS: 67-63-0, Carl Roth, Karlsruhe, Germany). Three μl of the test liquids (artificial saliva and diiodomethane) were placed on the specimen. This measurement was repeated 15 times. The samples were analyzed by using Young-Laplace shape fitting of the software (SCA20, DataPhysics Instruments GmbH, Filderstadt, Germany). All left and right corner CA’s were averaged (n = 30).

In contrast to literature, artificial saliva is not a common test liquid for a CA. It was chosen since saliva covered the oral cavity and is therefore essential for initial bacterial adhesion. Based on the measured CA, the surface free energy (SFE) was calculated by the OWRK method (Owens, Wendt, Rabel, Kaelble method) [25] using a software liquid database for diiodomethane and by our own experiments determined values for artificial saliva with: δ_l_ = 54.6 mN m^-1^, δ_l_^polar^ = 47.6 mN m^-1^, δ_l_^dispers^ = 6.98 mN m^-1^

SFE of artificial saliva was characterized by two reference solids (glass plate and PTFE plate) with standard testing liquids (diiodomethane and distilled water). As a second step the CA of this so far unknown artificial saliva was measured on the reference solids. SFE of the artificial saliva was derived by using OWRK´s approach. Consequently, it was possible to compare the results of the CA measurements to other studies by using the calculated SFE.

### 2.4 Microhardness

**Fig 1:**
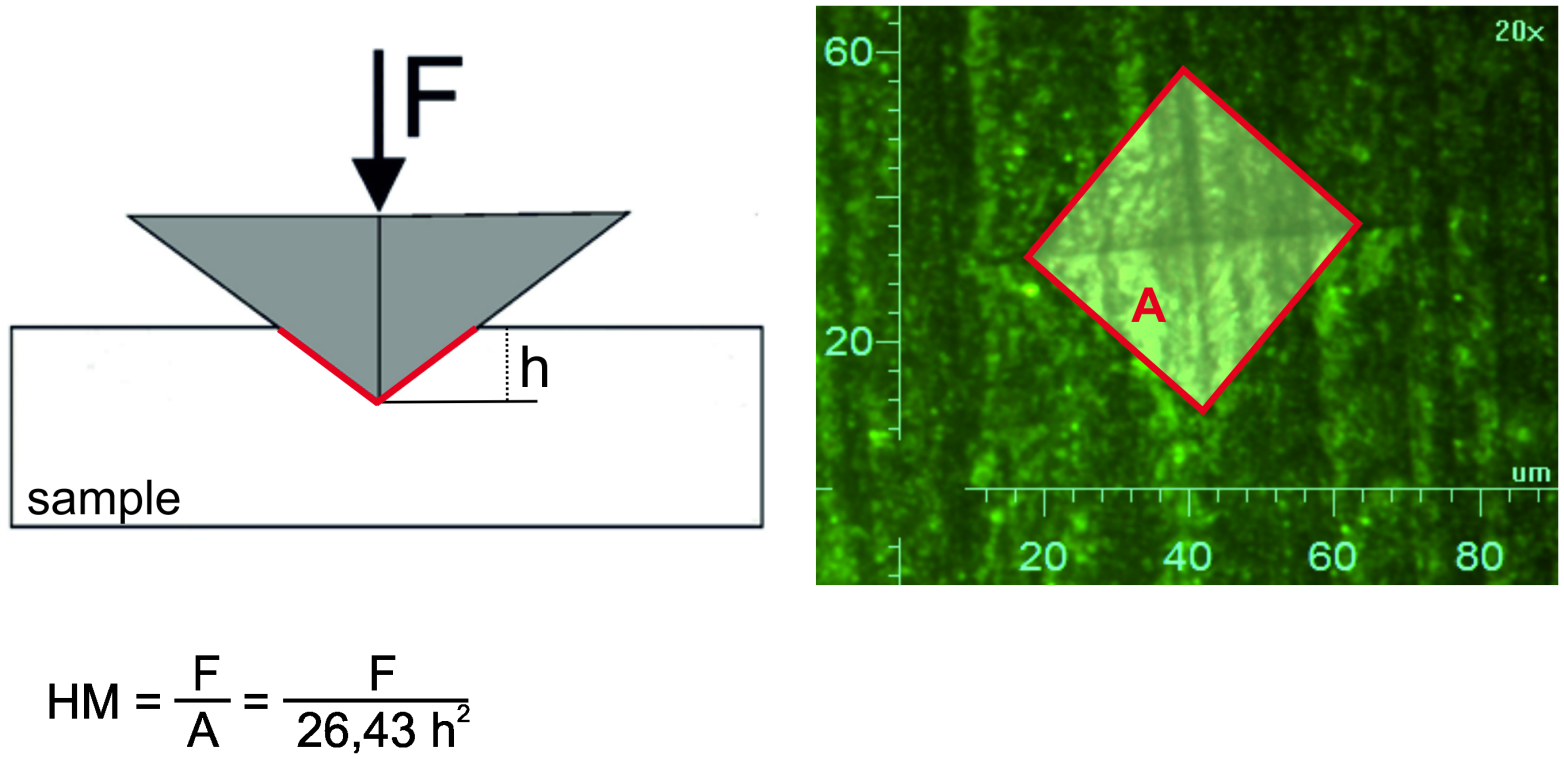
Measurement principle and calculation of Vickers microhardness.

Hardness represents a parameter for wear resistance and was measured with a Fischerscope HM 2000 (Helmut Fischer GmbH, Sindelfingen-Maichingen, Germany) equipped with a Vickers diamond indenter. By applying this instrumented indentation technique and continuously taking measurements of the penetration depth based on the applied force, a Martens hardness is measured and converted into a Vickers hardness (Fig 1). A maximum load of 1 N was applied with an indentation rate of 0.2 N s^-1^ and a holding time of 5 s at maximum load. Each sample was measured at 10 randomly chosen set points and the results were averaged.

### 2.5 Statistical analysis

All statistical calculations were carried out using SPSS 20.0 for Windows (SPSS Corporation, Chicago, IL, USA). Results were presented as median and interquartile range (IQR). CA and MHV were analyzed using the Kruskal-Wallis test for the three parameters (filler content, storage media and incubation time). A Post-hoc-analysis was performed to assess significant differences (*p < 0*.*05*) between these groups.

Correlation between SFE and time were examined by using bivariate correlation (Spearman Rank). All statistical tests were significant, if *p < 0*.*05*. Linear stepwise regression was carried out to analyze the absolute values of the CA and MHV at first and additionally the percental change to compare the influence of the parameters “incubation time”, “artificial aging media” and “composite” (filler content by weight). Pearson correlation was used to analyze which parameter correlated to CA or MHV. If *p < 0*.*05*, data were included into the regression model. The coefficients of determination *R*^*2*^ (range from 0 to 1) described the goodness of fit of the analyzed model, while the regression coefficient *β* was used to specify the influence of each parameter. In particular, the plus or minus signs for *β* need to be considered. This is explained in the following example: A minus sign of *β* means that the investigated value parameter e.g. CA is decreasing within the characterized parameter e.g. rising filler content. On the other hand a positive *β* means that CA is increasing within the characterized parameter.

## Results

### 3.1 Contact angle

CA was chosen as the parameter to describe the hydrophilic/hydrophobic characteristics and changes of the composites’ surface (Table 2).

**Table 2:** Contact angle (CA).

Composite		Low-filled (AF					Medium-filled (AT)					Highly-filled (G)				
Time (day)		0	14	30	90	180	0	14	30	90	180	0	14	30	90	180
storage media	AS	55,4 (12,9)	40,3** (9,2)	47,0 (9,5)	46,8 (7,5)	35,1** (10,8)	46,5 (10,5)	41,2 (17,8)	48,2 (9,4)	32,4* (7,8)	36,0 (6,6)	39,1 (6,9)	55,3* (7,9)	39,4 (5,8)	35,2 (11,8)	34,6 (3,9)
	LA pH 5	55,4 (12,9)	46,0* (6,5)	49,6 (5,8)	51,7 (9,4)	36,4* (10,5)	46,5 (10,5)	44,2 (8,5)	44,5 (8,7)	30,3** (5,7)	34,1* (6,4)	39,1 (6,9)	41,7 (2,7)	44,3 (7,3)	35,5 (7,9)	39,0 (3,3)
	LA pH 3	55,4 (12,9)	53,9 (4,0)	50,0 (5,9)	50,4 (6,7)	38,7** (12,4)	46,5 (10,5)	46,8 (6,4)	47,7 (8,8)	49,3 (10,5)	38,1 (8,9)	39,1 (6,9)	49,8* (8,3)	47,6* (3,7)	48,8* (14,7)	38,1 (6,2)
	CiA pH 5	55,4 (12,9)	50,9 (5,5)	53,4 (8,7)	53,0 (4,9)	39,8* (9,9)	46,5 (10,5)	51,1 (7,7)	43,4 (10,9)	39,9 (8,2)	38,5 (6,3)	39,1 (6,9)	39,2 (5,8)	45,0* (5,8)	42,8 (15,7)	40,1 (6,0)
	CiA pH 3	55,4 (12,9)	47,4* (4,5)	46,4* (8,1)	51,3 (11,1)	36,4** (10,5)	46,5 (10,5)	52,5 (7,2)	41,6 (9,4)	37,2 (4,6)	33,7* (6,6)	39,1 (6,9)	43,2 (13,6)	42,9 (5,8)	28,0 (13,6)	34,0 (6,4)
	Et 40%vol	55,4 (12,9)	46,7* (5,5)	51,4 (12,2)	57,4 (9,3)	35,6** (14,9)	46,5 (10,5)	40,4 (7,3)	48,3 (7,4)	38,1 (5,5)	33,4 (17,9)	39,1 (6,9)	38,9 (9,6)	41,1 (11,8)	41,6 (12,3)	33,8 (14,9)
	Et 60%vol	55,4 (12,9)	45,5* (6,1)	50,9 (6,2)	46,7* (8,9)	34,6** (11,9)	46,5 (10,5)	42,3 (6,0)	45,2 (9,6)	38,2 (10,3)	33,5* (8,2)	39,1 (6,9)	31,6* (4,8)	37,2 (5,5)	35,0 (14,6)	35,7 (6,2)

Medians (IQR) of the CA (°) measured with artificial saliva,

significant differences *(*p < 0*.*05)* and **(*p < 0*.*001)* to initial value (0 days) (Kruskal-Wallis-test);

grey filling (significant decrease or increase)

The CA of the initial state decreased with rising filler content from low-filled with 55.4°± 12.0 over medium-filled with 46.5°± 10.6 to highly-filled with 39.1° ± 6.0. Low-filled Arabesk Flow (AF) and medium-filled Arabesk Top (AT) showed significantly (*p < 0*.*05*) higher contact angles compared to the highly-filled GrandioSO (G). The difference between low-filled AF and medium-filled AT was not significant (*p = 0*.*318*).

The CA decreased for low-filled AF in all artificial aging media over time. The decrease was significant (*p < 0*.*05*) from initial 55.4° ± 12.0 up to maximal 34.6° ± 11.9 for ethanol 60%vol after 180 days. The medium-filled AT showed less decrease over time. Only LA pH 5, CiA pH 3 and ethanol 60%vol revealed a significant reduction (*p < 0*.*05*) of the CA after 180 days from 46.5° ± 10.5 down to a maximum of 33.5° ± 8.2. No significant decrease was proven for the highly-filled G after maximal incubation time. Instead a temporary increase (*p < 0*.*05*) of the CA and therefore a rise in hydrophobicity could be detected for the early and middle time periods (14 to 90 days) for AS, LA pH 3 and CiA pH 5. Only artificial aging with ethanol and especially under the higher ethanol concentration (60%vol) seemed to result in a reduced CA.

**Fig 2:**
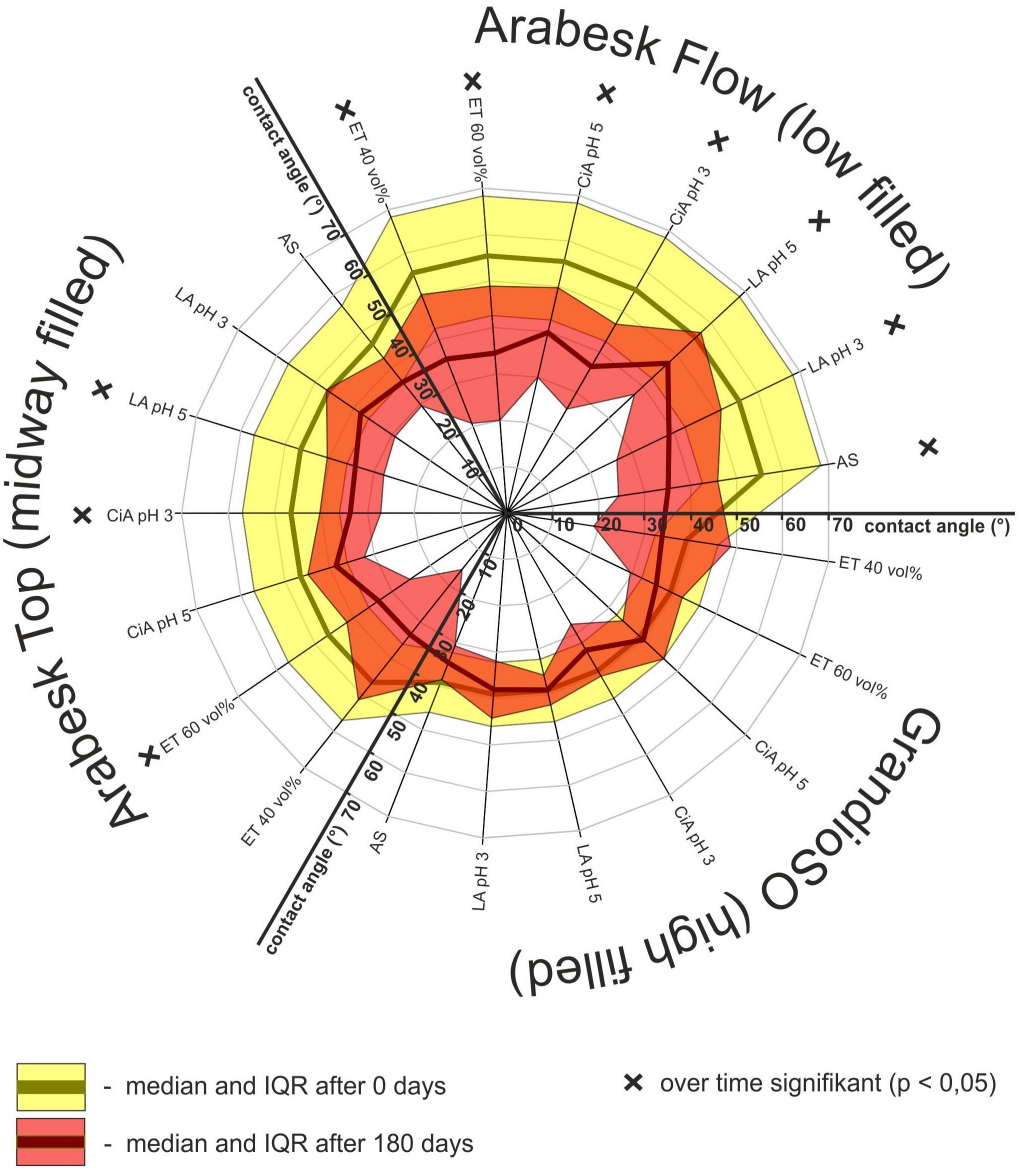
CA (sessile drop method; test liquid: artificial saliva) after 0 (yellow) and 180 (red) days.

To emphasize the influence of filler content solely on CA, the initial and final state were displayed in Fig 2. Differences between the initial state values of CA for the three composites are visualized together with the impact of filler content on the extent of CA reduction (Fig 2).

### 3.2 Surface free energy

The complex process of adhesion of bacteria on a surface e.g. composite surface, depends on, among other factors, the SFE. The approach used for the calculation of Owens, Wendt, Rabel, Kaelble assumed, that the SFE is the sum of the disperse part (London-van der Waals interactions) and polar interactions of permanent dipoles (Keesom) and induced dipoles (Debye).

Median values of the SFE as well as the ratio of polar to dispersive fraction (σ^p^/σ^d^) are shown in Table 3. Moreover, a bivariate correlation (Spearman rank) between SFE and time was carried out.

**Table 3:** SFE (%standard deviation) and ratio of polar/dispersive fraction (σ^p^/σ^d^).

Composite			Low-filled (AF					Medium-filled (AT)					Highly-filled (G)				
Time (day)			0	14	30	90	180	0	14	30	90	180	0	14	30	90	180
storage media	AS	SFE	46,2 (16,7)	53,7 (19,2)	51,9 (16,3)	54,0 (19,2)	57,2 (24,6)	52,3 (20,1)	54,5 (20,5)	51,3 (12,8)	57,7 (21,9)	55,8 (15,1)	52,6 (13,0)	51,2 (15,3)	53,9 (11,5)	57,0 (23,2)	56,2 (8,1)
		σ^p^/σ^d^	0,56	0,77	0,59	0,54	0,73	0,7	0,72	0,65	0,87	0,77	1,08	2,2	1,33	1,29	1,14
	LA pH 5	SFE	46,2 (16,7)	52,2 (8,1)	51,3 (10,3)	51,4 (13,7)	52,3 (25,4)	52,3 (20,1)	52,5 (12,1)	51,3 (16,0)	52,0 (15,5)	56,1 (19,3)	52,6 (13,0)	51,5 (9,6)	51,7 (11,0)	53,6 (23,5)	55,9 (8,4)
		σ^p^/σ^d^	0,56	0,67	0,58	0,57	0,65	0,7	0,7	0,68	0,84	0,79	1,08	1,38	1,48	1,32	1,26
	LA pH 3	SFE	46,2 (16,7)	49,5 (9,0)	51,8 (11,3)	52,3 (13,9)	56,0 (22,2)	52,3 (20,1)	53,7 (11,9)	53,5 (12,2)	58,8 (16,2)	56,9 (17,4)	52,6 (13,0)	54,7 (13,3)	52,9 (9,7)	56,9 (17,9)	55,1 (15,7)
		σ^p^/σ^d^	0,56	0,49	0,56	0,53	0,7	0,7	0,63	0,6	0,61	0,67	1,08	1,68	1,56	1,92	1,32
	CiA pH 5	SFE	46,2 (16,7)	50,2 (6,8)	49,9 (14,9)	52,2 (15,7)	55,6 (20,2)	52,3 (20,1)	48,6 (11,5)	54,5 (15,6)	56,4 (19,4)	57,5 (19,2)	52,6 (13,0)	51,7 (12,4)	52,7 (11,3)	59,3 (21,8)	57,1 (15,1)
		σ^p^/σ^d^	0,56	0,57	0,53	0,47	0,61	0,7	0,58	0,65	0,75	0,71	1,08	1,33	1,63	1,61	1,31
	CiA pH 3	SFE	46,2 (16,7)	52,3 (7,6)	52,7 (11,6)	53,4 (19,5)	57,3 (23,4)	52,3 (20,1)	50,8 (7,3)	53,2 (13,7)	54,5 (25,4)	55,9 (16,7)	52,6 (13,0)	54,7 (16,9)	53,6 (11,4)	52,5 (29,6)	53,8 (12,7)
		σ^p^/σ^d^	0,56	0,64	0,64	0,49	0,7	0,7	0,57	0,7	0,76	0,78	1,08	1,3	1,41	1,28	1,22
	Et 40%vol	SFE	46,2 (16,7)	52,8 (9,9)	48,6 (15,9)	50,7 (16,3)	55,2 (24,9)	52,3 (20,1)	53,9 (11,2)	53,2 (14,0)	56,4 (19,8)	56,2 (24,4)	52,6 (13,0)	53,6 (13,6)	53,1 (17,0)	53,8 (21,9)	53,9 (21,4)
		σ^p^/σ^d^	0,56	0,62	0,62	0,43	0,77	0,7	0,78	0,56	0,69	0,79	1,08	1,15	1,35	1,47	1,09
	Et 60%vol	SFE	46,2 (16,7)	51,8 (11,2)	49,5 (9,9)	52,0 (16,4)	55,0 (28,4)	52,3 (20,1)	53,6 (9,4)	53,8 (13,2)	53,4 (9,3)	57,2 (19,9)	52,6 (13,0)	56,3 (11,7)	55,2 (11,1)	56,5 (25,2	56,2 (15,3)
		σ^p^/σ^d^	0,56	0,66	0,57	0,66	0,77	0,7	0,73	0,64	0,72	0,84	1,08	1,03	1,19	1,31	1,23

**Fig 3:**
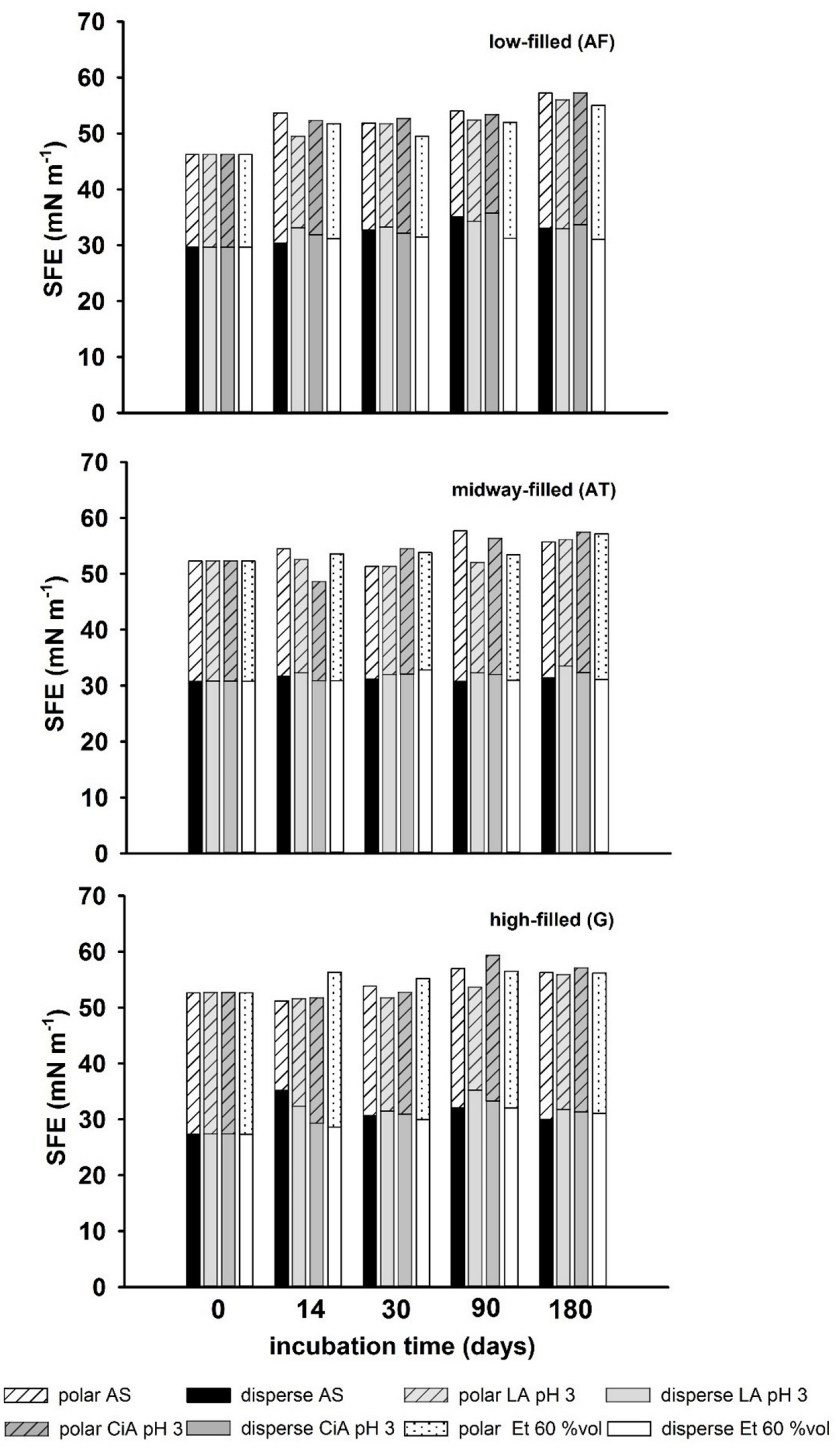
SFE with polar and disperse fractions over incubation time.

Initial values of SFE and the relation of the polar to dispersive fraction (σ^p^/σ^d^) varied between the composite materials. SFE increased with rising filler content from 46.2 mN m^-1^ (σ^p^/σ^d^ = 0.56) for low-filled AF up to 52.6 mN m^-1^ (σ^p^/σ^d^ = 1.08) for highly-filled G. Moreover, the relation of the polar to dispersive fraction (σ^p^/σ^d^) shifted with rising filler content from a quotient of 0.56 (AF) to 1.08 (G) towards the polar portion. The increase of SFE over incubation time reveals, that the surface became more hydrophilic. The highest increase was measured for low-filled AF and differed between the storage media with minimal changes of 13.2% for LA pH 5 and maximal changes of 23.9% under the influence of CiA pH 3. SFE showed a strong correlation depending on incubation time (*r*_*s*_ = *0*.*9*, *p < 0*.*05*). Medium-filled (AT) and highly-filled (G) composites showed less changes of SFE over time (Fig 3).

The increase of SFE for AT ranged from a minimum of 6.7% (AS) up to 9.9% (CiA pH 5) and for G from a minimum of 2.3% (CiA pH 3) up to a maximum of 8.6% (CiA pH 5). Correlation (Spearman rank) was demonstrated for some storage media. Significance was found between SFE and time (*r*_*s*_ = *0*.*9*, *p < 0*.*05*) for low-filled AF for all storage media, except LA pH 5 (*r*_*s*_ = *0*.*7*, *p = 0*.*188)* and Et 60%vol (*r*_*s*_ = *0*.*7*, *p = 0*.*188)*. Medium-filled AT revealed only for CiA pH 3 and pH 5 a significant correlation (*r*_*s*_ = *0*.*9*, *p < 0*.*05*) and for highly-filled G only one strong correlation (*r*_*s*_ = *0*.*9*, *p < 0*.*05*) was found for Et 40%vol. However, based on the comparison of the ratio of polar to dispersive fractions (σ^p^/σ^d^) the increase of SFE over incubation time is predominantly influenced by the increase of polar fraction, which was demonstrated for all investigated composites. Storage in ethanol 60%vol showed the highest shift of the ratio of polar to disperse fractions (σ^p^/σ^d^) from 0.56 up to 0.77 for AF and from 0.7 up to 0.84 for AT. Also, highly-filled composite G revealed high shifts of σ^p^/σ^d^ for all storage media and a maximum change under storage in LA from 1.08 up to 1.32.

**Fig 4:**
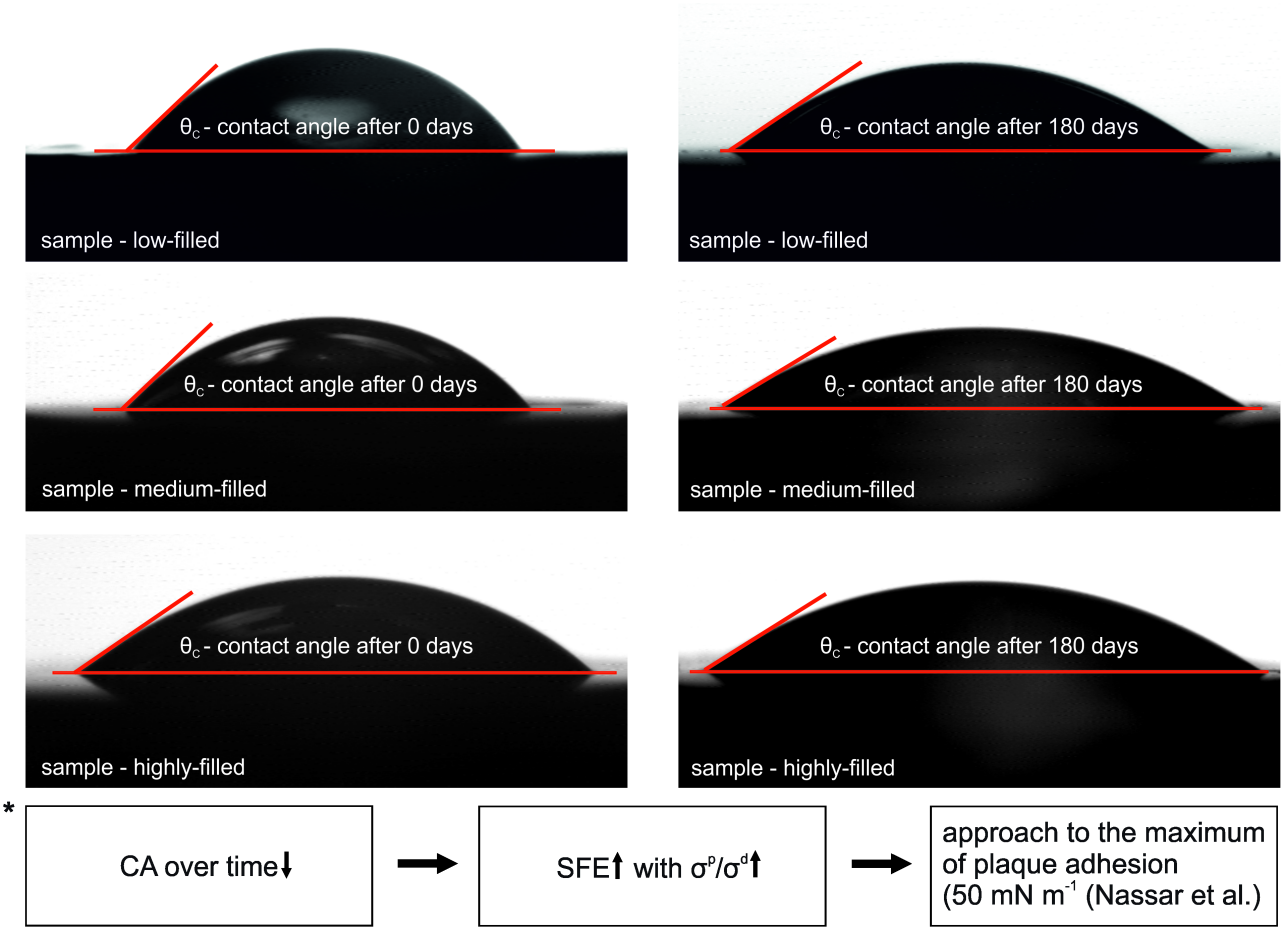
Contact angle (CA) measurement: Exemplary pictures of droplets of all composites for artificial saliva at 0 and 180 days; * estimation of bacterial adhesion according to the threshold value of 50 mN m^-1^ [26].

The measurement principle is shown exemplary for all three composites for the test liquid artificial saliva (Fig 4). It was obvious that a lower CA resulted in a rising SFE and accordingly to the know literature a higher plaque adhesion can be expected until a maximum of 50 mN m^-1^ [26].

### 3.3 Vickers microhardness (MHV)

The changes of MHV of all tested composites are shown in Table 4. Considerable differences existed for the initial MHV values between low-filled, medium-filled and highly-filled composites (*p < 0*.*05*) ranging from 44.7 ± 3.9 MHV up to 124.9 ± 44.8 MHV. Values differed by a factor of 2.8 and changed over the maximum incubation time (180 days).

**Table 4:** Microhardness (MHV).

Composite		Low-filled (AF					Medium-filled (AT)					Highly-filled (G)				
Time (day)		0	14	30	90	180	0	14	30	90	180	0	14	30	90	180
storage media	AS	44,7 (3,9)	50,5 (4,2)	49,9 (7,7)	50,1 (9,5)	49,3 (8,9)	58,9 (8,7)	54,9 (9,0)	47,6 (21,6)	63,3 (9,2)	54,0 (11,6)	124,9 (44,8)	129,6 (36,5)	130,1 (14,8)	129,0 (25,1)	110,6 (52,7)
	LA pH 5	44,7 (3,9)	50,4* (3,0)	49,8 (4,1)	46,0 (5,1)	48,7 (9,3)	58,9 (8,7)	50,4 (4,5)	54,2 (14,4)	61,4 (11,1)	45,1* (15,6)	124,9 (44,8)	127,8 (16,6)	130,5 (37,4)	105,7 (61,4)	127,6 (21,4)
	LA pH 3	44,7 (3,9)	49,6* (2,5)	48,6 (10,4)	48,1 (2,5)	49,5 (4,1)	58,9 (8,7)	38,1* (7,2)	52,3 (10,0)	64,6 (7,5)	63,7 (5,1)	124,9 (44,8)	118,3 (29,7)	147,1 (25,9)	123,8 (31,8)	115,4 (32,0)
	CiA pH 5	44,7 (3,9)	40,0 (8,0)	49,0 (5,3)	52,0 (8,9)	50,8 (8,5)	58,9 (8,7)	44,9 (10,6)	59,8 (10,8)	63,8 (10,7)	61,0 (10,3)	124,9 (44,8)	119,5 (14,4)	124,6 (23,3)	124,9 (14,4)	126,3 (18,0)
	CiA pH 3	44,7 (3,9)	28,9* (9,6)	47,6 (2,9)	33,0 (4,3)	48,5 (11,8)	58,9 (8,7)	54,5 (7,7)	58,7 (6,9)	64,0 (8,2)	40,7 (29,7)	124,9 (44,8)	133,2 (69,9)	117,2 (40,7)	129,8 (14,3)	111,1 (32,7)
	Et 40%vol	44,7 (3,9)	41,4 (10,1)	45,3 (5,0)	37,6* (5,0)	33,0* (5,0)	58,9 (8,7)	55,3 (6,4)	47,2* (8,4)	51,3 (2,4)	40,4** (12,4)	124,9 (44,8)	120,9 (23,3)	116,1 (28,8)	105,7 (14,5)	101,1* (16,0)
	Et 60%vol	44,7 (3,9)	43,9 (8,5)	43,1 (5,7)	38,6* (3,2)	34,2* (8,2)	58,9 (8,7)	54,3 (12,2)	47,9 (5,9)	39,2** (3,9)	42,6* (5,2)	124,9 (44,8)	115,5 (13,5)	113,4 (10,9)	108,9 (44,4)	107,6 (54,6)

Medians (IQR) of the microhardness (MHV)

significant differences *(*p < 0*.*05)* and **(*p < 0*.*001)* to initial value (0 days) (Kruskal-Wallis test); grey filling (significant decrease or increase)

Changes of MHV are mostly limited to artificial aging with ethanol (40%vol and 60%vol). Significant decreases were especially demonstrated with low-filled AF (*p < 0*.*05*) from initial 44.7 ± 3.9 MHV to 37.6 ± 5.0 MHV after 90 days and 33.0 ± 5.0 MHV after 180 days under storage in ethanol 40%vol. A similar decrease was revealed under influence of ethanol 60%vol from initial 44.7 ± 3.9 MHV to 38.6 ± 3.2 MHV after 90 days and 34.2 ± 8.2 MHV after 180 days. All other storage media (AS, LA and CiA) led only to a slight increase of hardness, which was not significant.

The results for medium-filled AT had a negligible difference compared to low-filled AF. Decreases from an initial 58.9 ± 8.7 MHV to 40.4 ± 12.4 MHV (40%vol) and 42.6 ± 5.2 MHV (60%vol) were detected after 180 days (both ethanol concentrations), additionally the ethanol 60%vol concentration decreased the MHV already after 90 days. Incubation under any other artificial aging media showed no significant changes (*p > 0*.*05*) except LA pH 5 (180 days) and CiA pH 3 (14 days).

MHV of the highly-filled G decreased while stored in ethanol, but only showed significant change when stored in ethanol 40%vol after 180 days.

### 3.4 Regression analysis for modeling most influential parameters

The influence of the variables (incubation time, storage media and filler content) was analyzed using linear regression models for the CA and the MHV for the absolute values as well as the percental change, which was chosen for comparison of relative changes to initial state.

The regression analysis of absolute values (Table 5) depicts the influence of incubation time and filler content. Storage media was not included into the CA model due to missing Pearson correlation. The corrected *R*^*2*^, which consider compared to the normal *R*^*2*^ the number of included variables, of 0.196 was not highly developed. The standard error (7.716) can be used to estimate the distribution of the single values referring to the regression line. Based on a Pearson correlation, the variable’s filler content and incubation time were included into the regression model and reveal equal standardized regression coefficients *β* of -0.315. The negative *β* shows that a rising filler content and an increasing incubation time led to a decrease of CA and the surface becomes more hydrophilic.

**Table 5:** Stepwise linear regression of the absolute value of CA.

	model summary		coefficients		sig.
	corrected R2	standard error of the estimator	regression coefficient (B)	standardized regression coefficient (beta)	
	0.196	7.716			
(constant)			57.630		0.000
filler content			-3.322	-0.315	0.000
incubation time			-2.265	-0.313	0.000

The regression analysis of the percental change of the CA in relation to initial state (baseline) was assessed to assess the influence of the variables on the extent of the degradation. The change of the CA depending on incubation time and filler content could be proven by the results of stepwise linear regression (Table 6). The corrected *R*^*2*^ of 0.274 is not highly developed. Due to the positive *β* of 0.429 for filler content, it can be concluded, that rising filler content led to less change of CA over incubation time. In contrast, a longer incubation time increased the percental change with a negative *β* of -0.299.

**Table 6:** Stepwise linear regression of the percental change (Δ%) of the CA.

	model summary		coefficients		sig.
	corrected R^2^	standard error of the estimator	regression coefficient (B)	standardized regression coefficient (beta)	
	0.274	16.845			
(constant)			-6.544		0.003
filler content			10.392	0.429	0.000
incubation time			-5.278	-0.299	0.000

In contrast to the analysis of the CA, the linear regression model for hardness included artificial aging media and filler content. Since the MHV was differing for the initial state among the tested composites, the regression analysis of absolute values showed a relatively strong linear relation with a corrected *R*^*2*^ of 0.676 (Table 7).

**Table 7:** Stepwise linear regression of the absolute value of MHV.

	model summary		coefficients		sig.
	corrected R^2^	standard error of the estimator	regression coefficient (B)	standardized regression coefficient (beta)	
	0.676	21.296			
(constant)			4.049		0.099
filler content			37.472	0.816	0.000
artificial aging media			-1.699	-0.093	0.000

The positive *β* of 0.816 of the variable filler content suggests that an increased filler content leads to a higher MHV. With a *β* of 0.816 the filler content was the predominant variable influencing MHV. In comparison, artificial aging media with its impact based upon a low *β* of -0.093 as well as incubation time and storage medie which were not included into the model due to missing correlation, can be neglected.

The regression analysis of the percental change showed a much lower corrected *R*^*2*^ of 0.172 (Table 8). In contrast to the regression of the absolute values, filler content had a negative *β* of -0.379, which indicates that with rising filler content, degradation had less effect. In comparison to filler content, the influence of artificial aging media (with a *β* of -0.177) on the extent of degradation was of minor importance.

**Table 8:** Stepwise linear regression of the percental change (Δ%) of MHV.

	model summary		coefficients		sig.
	corrected R2	standard error of the estimator	regression coefficient (B)	standardized regression coefficient (beta)	
	0.172	38.696			
(constant)			35.903		0.121
filler content			-19.808	-0.379	0.000
artificial aging media			-3.774	-0.177	0.000

## Discussion

Due to their chemical composition, the aging of composites in the oral cavity is unavoidable. Both, chemical hydrolysis and material properties determine the extent of degradation. Thus, three composites of different filler content were investigated over an incubation time of 6 months in several artificial aging media. This study was focused on chemical hydrolysis and its effects on the composite surface characteristics.

To study the complex process of artificial aging in the oral cavity, chemical, mechanical and thermal approaches were utilized in the literature. Every experimental study should aim towards the most realistic circumstances, which is almost impossible to achieve with today’s techniques and proportional expenses. Various research was done on short term effects (up to 30 days) and focused on leaching the residual monomer content [27, 28, 29, 13]. The rate and extent of eluted monomers depend on the storage media, the structure of the composite and the conversion rate. Ethanol (e.g. 75%vol) as well as other acetic aqueous solutions like lactic, acetic or propionic acids are recommended as a storage media due to their high swelling capacity (ethanol and acetic aqueous solutions) and hydrophobicity (especially ethanol) [28]. For long-term studies, which focus more on the chemical breakdown, the incubation time seems to be more important than the capability of elution. In comparison to various short time studies (up to 30 days), only a few studies exist with a longer incubation duration without using accelerated conditions like thermocycling [30, 19, 31, 11, 32].

However, the aim of this study was to apply a method using lower ethanol concentrations (comparable to more lipophilic media) and acidic aging media over an extended incubation time with regards to the filler content.

The important influence of filler content on the initial state of the CA and MHV was shown in several other studies [33, 34, 35, 36, 37]. MHV has been reported to range from 270 to 420 MHV for enamel and from 10 to 90 MHV for dentin [38, 39]. All three composites were much softer than the antagonist cusps. Only dentin was in the range of the investigated composites MHV. Thus, abrasion loss will be located mainly on the composite restoration [40].

Also, the initial state of the CA according to that of SFE was found to depend on filler content. Rising filler content led to a lower CA of polar liquids like the artificial saliva used. Due to the decreasing CA the SFE and primarily the polar fraction (Keesom- and Derby energy) of SFE increased with rising filler content. Bürgers et al. [41] found similar relations for various fissure sealants, which can be classified as low-filled composites.

The higher relation of exposed fillers with high SFE compared to the resin matrix with low SFE on the surface could explain the higher SFE and polar fraction, which was found in this study.

It has to be expected, that the low CA of AS and the resulting high SFE will cause high plaque adhesion by oral bacteria species like *Streptococcus mitis*, *Streptococcus oralis*, *Streptococcus sanguis*, *Actinomyces naeslundii und Actinomyces viscosus* [26, 42, 43]. However, the results of this study revealed even at the initial state a high SFE with 46.2 mN m^-1^ up to 52.6 mN m^-1^, which are close to the from Nassar et al. defined maximum of 50 mN m^-1^ [26]. Even if studies [6, 44] showed, that roughness is more important for plaque adhesion than SFE, there was still a dependence especially for early plaque adhesion. This influence was proven by an experimental study using surface modifications (polymerizable active agent, e.g. silicone polyether acrylate), which reduced the total SFE and polar fraction and led to a reduced amount of early bacterial colonization [43, 45]. Further experiments of ours showed that there were no significant changes of roughness measured by confocal laser scanning microscopy after the same artificial aging protocol, which was used in this study. All composites used in this study were below a known threshold of 0.2 μm [46, 47] before and after artificial aging. Thus for clinical performance, changes of SFE might be more important for these particular composites than changes of roughness.

Our own results supported the findings of other studies, which describe a decline of mechanical and surface properties by chemical degradation using common liquids of the oral cavity [30, 18, 21, 48, 32]. This reduction of properties like hardness, roughness, SFE will also worsen their clinical performance over time. Considering that recent composite restorations have a long operational lifespan (annual failure rates: 2.4% in 10 years), constant material properties are necessary to maintain good clinical performance [7].

There are just a few studies characterizing the CA under different artificial aging conditions. One research group found a significant (*p < 0*.*05*) increase of the CA while stored for one year in water, artificial saliva and ethanol (96%vol), as well as a decrease (*p < 0*.*05*) when using thermocycling with distilled water [18, 41]. These results were partially contrary to the findings of our study. One main difference regarding the methods utilized in our study beside different storage media, was the drying procedure, which was carried out after artificial aging and before measuring. The influence of absorbed liquids on the CA is well known and described in literature [49, 50, 51]. A possible explanation of a decreased CA is that superficial matrix connections are split and matrix components are leached. In consequence, the ratio of filler to matrix surface will shift to a more polar filler dominated surface. The increase of the polar fraction part of SFE and the shift of the polar/disperse ratio supported this assumption. It was obvious that future studies will be necessary to describe the process in detail.

The quantity of artificial aging depends on filler content and incubation time. Hence, the maximum decrease can be observed after a maximum aging duration of 180 days. Therefore, a longer incubation time should be considered. The influence filler content had on the initial state ran in contrary to the influence it had on the quantity of artificial aging. While rising filler content decreased the initial CA, the magnitude of change was less distinctive. Higher filler content led to reduced water absorption [10, 52], consequently the effective artificial aging media volume, which was absorbed, is also reduced, which led to less chemical stress [53]. Another study with experimental composites supported our results by measuring the amount of leached degradation products, which was reduced when using highly-filled composites [54]. As a consequence, the lower initial CA predominate the reduced degradation in this study, so a higher plaque adhesion could be expected. If it is possible to improve the initial CA of highly-filled composites for example by surface modifications as shown by Rüttermann [43], the reduced SFE and the reduced changes over incubation time could be beneficial.

A common problem when measuring CA, is the strong influence of the test environment (e.g. temperature and air moisture). It should be considered to use an environmental chamber for surface characterization. In general, it must be mentioned, that all three composites featured a high SFE even at initial state. A maximum adhesion for oral bacteria species was found for a SFE from 45 to 55 mN m^-1^ [26]. The minimum level of this range was reached by each of the assessed composites. However, artificial aging led to even higher SFE and consequently probably to a higher plaque adhesion. This adhesion could be characterized directly using fluorescence microscopy for example [18, 41].

The comparison of the results concerning MHV to other studies appears to be quite difficult. The main problem is the use of differing measuring methods and the strong influence of the applied forces and holding time, which are often not even mentioned precisely. The most common hardness testing methods are Knoop hardness using a typical load of 50g and 15s holding time and Vickers hardness, where various loads (ranging from 100g up to 500g) and holding times (ranging from 5s up to 60s) are used. Storage in ethanol decreased results (irrespective of Knoop hardness or Vickers hardness) in most studies [13, 55, 11, 19, 56, 30]. This can be confirmed by our data. MHV decreased under artificial aging with ethanol for all three composites, but changes of low-filled AF and medium-filled AT were significant (*p < 0*.*05*) after just 30 and 90 days of storage. Highly-filled composite G revealed a significant decrease (*p < 0*.*05*) only after the maximum time of 180 days. Similar results were found in most studies for ethanol solutions, while studies with different acidic media showed diverse results [11, 57, 32, 58, 59, 60].

Studies, that assessed hardness changes (mainly Knoop hardness testing) after storage (28 up to 180 days) in acidic drinks like fruit juices or so called energy drinks reported a higher decrease of hardness values compared to a control group of distilled water [58, 32, 60].

Other studies, which used lactic and citric acids for artificial aging showed a decrease of hardness values (Knoop hardness testing) for most tested composites under the influence of lactic acid [11, 56, 57, 59]. The decrease seemed to be material dependent. Citric acid showed different results with no effect on hardness values or even an increase of hardness after storage (up to 1 year) [56, 11], while another study with a pH sequence of citric acid ranging from 2.5 up to 7 revealed a pH depending decrease already after two weeks of artificial aging [59].

In agreement with previously published studies, the results of this study indicate, that changes of hardness values due to acidic liquids depended on material and concentration, but lacking consistent rules. While low-filled AF showed a slight, but not significant increase after maximum storage of 180 days in both lactic and citric acid, medium-filled AT and highly-filled G revealed increases and decreases, which were not significant with one exception under influence of LA pH 5. Differences to other studies, which found pH based artificial aging with various acid and food ingredients, could exist due to different artificial aging protocols like a 24h buffering in distilled water before initial hardness testing to extract most of the residual monomers. Moreover, the method (Knoop and Vickers hardness) and the relevant parameters of hardness testing like load or holding time vary between most studies [61, 62, 63]. Furthermore, hardness testing depends on water absorption. That means a higher water sorption may lead to a lower hardness. Unfortunately, some studies do not describe these parameters or their experimental setup is not comparable to our own experiments.

In addition to statistical analysis using Kruskal-Wallis tests the linear regression model helped to discuss the influence of the variables (incubation time, storage media and filler content) on the extent of the artificial aging for the CA and the MHV separately.

The results of linear regression supported the demand of highly-filled composites to minimize abrasion. Even if the highly-filled G showed the highest percental changes (β of filler content = -0.379), regression of absolute values indicated that the MHV increased strongly with rising filler content.

It should be considered that the influence of silanized fillers which are used in G cannot be separated from the overall influence of filler content based on the way this study was designed. Other studies showed significant increase of hardness with silanized fillers [64], [65]. This could be shown with our own results of the initial state for the highly-filled G with 89 wt% and a MHV of 124.9. One explanation for the higher percental change of hardness of G could be a breakdown of silane bonds, which was already described in literature [21]. Further investigations to separate the general influence of filler content and silanization are recommended.

The considerable larger variance (increased by a factor of 5) of hardness values of G compared to the other composites might be explained by an inhomogeneous hardness distribution, based on the diverse filler composition. In consequence of the used instrumented indention method measuring points were placed arbitrarily. Thus, it is not clear, whether the indention was made on a filler or the matrix areal. Whether this inhomogeneous distribution led to accelerated abrasion under mechanical loading [8], could not be discerned with the aging protocol used. Further investigations using a chewing simulator for example or under *in vivo* conditions are necessary.

As a consequence of the reduced hardness and increased hydrophilic wetting behavior after artificial aging it can be assumed that over the operational lifespan of a composite the properties of the restoration might decline. This will become manifest in reduced wear resistance and slightly increased plaque adhesion. Nevertheless, through permanent abrasion the affected surface areas will be removed continuously and expose fresh areas. Filler content seemed to be the most important variable, while artificial aging media and artificial aging time played a minor role. Thus, low-filled composites used on occlusal surfaces and especially low-filled fissure sealants, which are often used in pediatric dentistry, should be monitored regularly. It could be shown, that ethanol is an efficient artificial aging media even in a lower concentration of 40 and 60%vol, while fruit acids and acids derived from bacteria could mainly affect CA [56, 11, 18].

Beside the influence on hardness, filler content and the monomer matrix system play a key role in reducing the polymerization shrinkage (range of 1.5–5%) and the associated shrinkage stress when restrained to a cavity surface [1, 4, 66]. In which extent changes of shrinkage stress occurred under water absorption *in vivo* over time and this reversed effect might be used in advantage is not satisfyingly investigated [66, 67]. However, it is not being proven, whether high polymerization shrinkage and stress even caused shorter longevity [68, 69].

In general, the results of this *in vitro* study are supported by the findings of other authors. Ethanol as a capable artificial aging media showed significant decrease of MHV, while LA and CiA revealed diverse results. CA decreased over incubation time under the influence of all storage media, while the magnitude of change depended on the filler content. The hypothesis can be supported that filler content, incubation time and artificial aging media are important variables for the quantification of proceeded artificial aging.

Due to the limitation of the artificial aging model used, it must be expected, that degradation under oral conditions will be more pronounced. Enzymes like cholesterol esterase and pseudo-cholinesterase led to a catalytic breakdown on matrix bonds [20, 70, 71, 23] and should be considered further. In addition, the omnipresent pellicle on every oral surface and the dynamic saliva flow cannot be simulated. These additional influences should be assessed by executed in an *in vivo* study.

## Conclusion

During the long-lasting function of dental composites inside the oral cavity significant changes on the mechanical and physico-chemical properties should be expected. Composite material and especially rising filler content seemed to improve the materials’ resistance against chemical degradation processes, while incubation time and artificial aging media were less important. For ethanol it could be found, that it is an effective artificial aging media and that it causes distinctive changes compared to acids especially for MHV. Because of the decreased surface properties, the clinical performance concerning wear resistance and plaque adhesion will decline, too.
